# MicroRNA-26a inhibits the growth and invasiveness of malignant melanoma and directly targets on *MITF* gene

**DOI:** 10.1038/cddiscovery.2017.28

**Published:** 2017-07-10

**Authors:** Hui Qian, Chen Yang, Yixin Yang

**Affiliations:** 1 Department of Biological Sciences, Emporia State University, Emporia, KS, USA

## Abstract

Metastatic melanoma is the most aggressive form of skin cancer and is refractory to therapy. MicroRNAs have been recently discovered as novel molecules that provide therapeutic benefits against melanoma. This work aims to examine the effects of miR-26a and let-7a on the growth and invasiveness of malignant melanoma *in vitro* and *in vivo*. In addition, we elucidate the mechanism of action by identifying the target gene of miR-26a. Both miR-26a and let-7a inhibited proliferation and invasiveness and halted the cell cycle at the G_1_/G_0_ phase in SKMEL-28 and WM1552C malignant melanoma cell lines. Moreover, miR-26a potently induced apoptosis and downregulated the expressions of *microphthalmia-associated transcription factor (MITF)* and MAP4K3 in both cell lines. The luciferase reporter assay demonstrated that miR-26a suppresses MITF expression by binding the 3′-UTR, suggesting that MITF is a *bona fide* target of miR-26a. SiRNA knockdown of the *MITF* gene confirmed that miR-26a reduced cell viability and induced apoptosis by regulating MITF. Using a murine model, we also found miR-26a significantly retarded the growth of melanoma tumors *in vivo*. In conclusion, miR-26a and let-7a suppressed the growth and invasiveness of melanoma cells, suggesting that miR-26a and let-7a may represent novel therapies for malignant melanoma.

## Introduction

The incidence of metastatic melanoma, the deadliest skin cancer, has increased in the past two decades. It is estimated that 76 380 new melanoma cases will be diagnosed in 2016.^1^ The prognosis of melanoma is poor, with the lower than 20% of 5-year survival rates for patients who harbor metastatic melanoma.^2,3^ Since about 50% patients with melanoma have been found to harbor the activating BRAF^V600E^ mutation,^4^ the current targeted therapy focuses on RAS-RAF-MEK-ERK signaling pathway.^5^ Even though the initial response to BRAF inhibitors (vemurafenib and dabrafenib) and MEK inhibitors (trametinib and cobimetinib) is impressive, resistance usually develops in the patients within 6–8 months.^6^ Thus, looking for alternative effective therapies for the metastasized melanoma is an imperative task.

MicroRNAs (miRNAs) are engaged in post-transcriptional regulation of gene expression by directly targeting the 3′-UTR of mRNA.^7^ Among the microRNAs that are implicated in tumor growth and progression, miR-26a is involved in a variety of signaling pathways and can act as a tumor suppressor in carcinogenesis and cancer progression.^8^ Previous studies found that miR-26a inhibits the proliferation of gallbladder cancer cells and breast cancer cells by downregulating high-mobility group AT-hook 2 (HMGA2) and MCL-1 expression, respectively.^9,10^ In addition, enhancer of zeste homolog 2 was also directly regulated by miR-26a, leading to the inhibition of migration and invasion in osteosarcoma,^11^ and tumorigenesis repression in nasopharyngeal cancer.^12^ It has been identified that the expression of miR-26a is specifically downregulated in human melanoma cells.^13^ It was reported that miR-26a induced apoptosis of melanoma cells by directly targeting the silencer of death domains (SODDs), which has a vital role in preventing apoptosis in the sensitive cell lines of melanoma.^13^

Let-7a acts as tumor suppressor microRNA by negatively regulating the expressions of RAS,^14^ HMGA,^14^ C-MYC,^15^ and NIRF^16^ oncogenes in lung cancer, renal cell carcinoma, and colorectal cancer, respectively. Let-7a is also involved in many important signaling pathways in cancer progression.^17,18^ Furthermore, let-7a induces apoptosis in a number of cancers including gastric carcinoma,^19^ nasopharyngeal carcinoma,^20^ hepatocellular cancer,^21^ breast cancer,^22^ cervical cancer,^23^ brain cancer,^24^ and prostate cancer.^25^ For melanoma, let-7a was found to reduce invasive potential of melanoma cells by regulating the expression of integrin-β3.^26^ In addition, let-7a increased melanoma cell sensitivity to doxorubicin.^27^ Although the tumor suppressor functions of let-7a in other types of cancers is well documented, little research has been done on investigating the inhibitory effects of let-7a on the growth properties of malignant melanoma cells. The biological role of let-7a remains largely unknown in human malignant melanoma.

This study characterizes the tumor-suppressing effects of let-7a and miR-26a in malignant melanoma and identifies, for the first time, *microphthalmia-associated transcription factor (MITF*) as a novel gene target of miR-26a in malignant melanoma. In addition, this study is the first work that examines the inhibitory effect of miR-26a on melanoma growth in mice. This work establishes that miR-26a replacement is a potential therapeutic strategy for malignant melanoma.

## Results

### Transfection of miR-26a and let-7a mimics significantly decreased cell viability in human malignant melanoma cell lines

**Figure 1:**
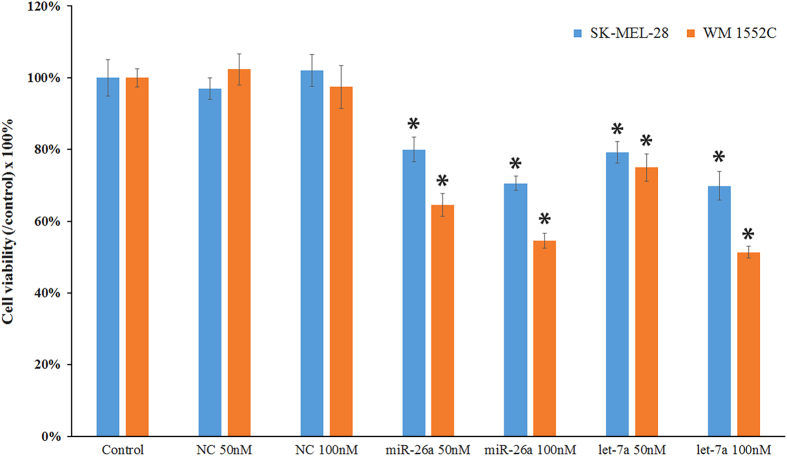
The effect of miR-26a and let-7a on the cell viability of SKMEL-28 and WM1552C melanoma cells. Cell viability was determined using the MTT assay after 48 h transfection with transfection reagent alone (control), AllStars negative control siRNA (100 nM), miR-26a mimics, and let-7a mimics at a final concentration of 50 or 100 nM. Each experiment was repeated six times and the results are presented as mean±S.D. Asterisks indicate a significant difference (*P*<0.05) compared with control groups.

The expression of miR-26a and let-7a was detected by qRT-PCR in SKMEL-28 and WM1552C melanoma cell lines. The expression levels of let-7a in both cell lines were very low, especially in WM1552C cell line (data not shown). The data did not support our prediction that the expressions of miR-26a and let-7a were lower in SKMEL-28 cells, which was derived from a melanoma of more advanced stage than that of WM1552C cells. Transfection of miR-26a elevated the level of miR-26a by 511.4- and 1462.2-fold in SKMEL-28 and WM1552C melanoma cells, respectively. Similarly, transfection of let-7a mimics elevated the level of let-7a by 1535.5- and 4733.3-fold in SKMEL-28 and WM1552C melanoma cells, respectively. Transfection of miR-26a or let-7a significantly reduced the viability of SKMEL-28 and WM1552C melanoma cell lines (Figure 1). SKMEL-28 is more resistant to the cytotoxicity of miR-26a and let-7a compared with WM1552C.

### MiR-26a and let-7a inhibited cell migration and invasion

**Figure 2:**
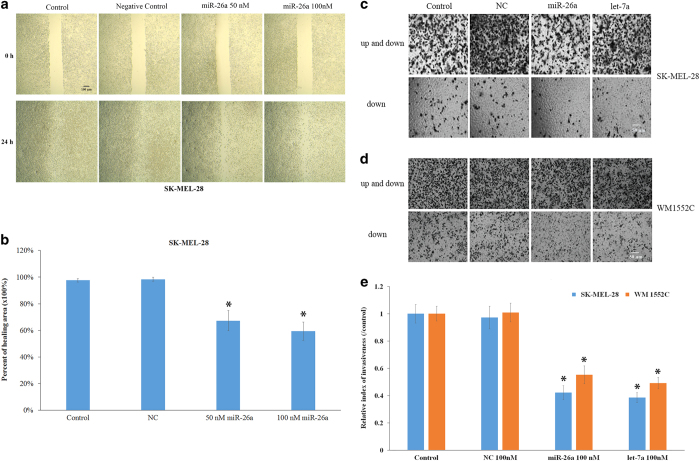
MiR-26a inhibited cell migration and invasion. (**a** and **b**) After 48 h transfection of negative control microRNA mimics or miR-26a mimics in SKMEL-28 cells at the final concentration of 50 or 100 nM, the cell migration assay was performed as described in the Materials and Methods section. The area of the wound was evaluated under a microscope (×100) and determined using the Image J software (National Institutes of Health, Bethesda, MD, USA). The inhibition of cell migration was measured with the following formula: the percent of healing area=(wound area at 0 h–wound area at 24 h)/(wound area at 0 h). SKMEL-28 (**c**) and WM1552C (**d**) melanoma cells were seeded into chambers with serum-free medium after transfection of miR-26a or let-7a at a concentration of 100 nM for 48 h. The chambers were inserted into a 24-well plate with medium containing FBS, and the reduced cell invasion ability was detected by the number of cells located on the bottom side of the chamber observed by microscope (×400) after 24 h incubation. The relative invasive index of each group is presented in a bar graph (**e**). Experiments were repeated three times independently and the results are presented as the mean±S.D. Asterisks indicate a significant difference (*P*<0.05) compared with control groups.

We further examined the effects of miR-26a on cell migration ability that was detected by the wound healing assay. SKMEL-28 melanoma cells that were transfected with 50 or 100 nM miR-26a showed a decreased ability to close the wound compared with untreated cells or cells transfected with negative control mimics, suggesting that miR-26a significantly inhibited cell migration (Figures 2a and b). The microRNA let-7a at 100 nM also displayed a strong inhibitory effect on cell migration (Supplementary Figure 1).

In addition, the effect of miR-26a and let-7a on cell invasion ability, which is a hallmark of malignancy, was examined using a Transwell assay. MiR-26a and let-7a significantly (*P*<0.05) reduced the cell invasiveness in both SKMEL-28 and WM1552C melanoma cell lines (Figure 2). MiR-26a and let-7a (100 nM) suppressed cell invasion in SKMEL-28 cells by 57% and 61%, respectively (Figure 2c). In the WM1552C cell line, miR-26a and let-7a inhibited the cell invasion by 44% and 51%, respectively (Figure 2d). Therefore, both miR-26a and let-7a significantly suppressed the migration and invasiveness of melanoma cells.

### MiR-26a and let-7a caused the cell cycle to halt at the G_1_/G_0_ phase

**Figure 3:**
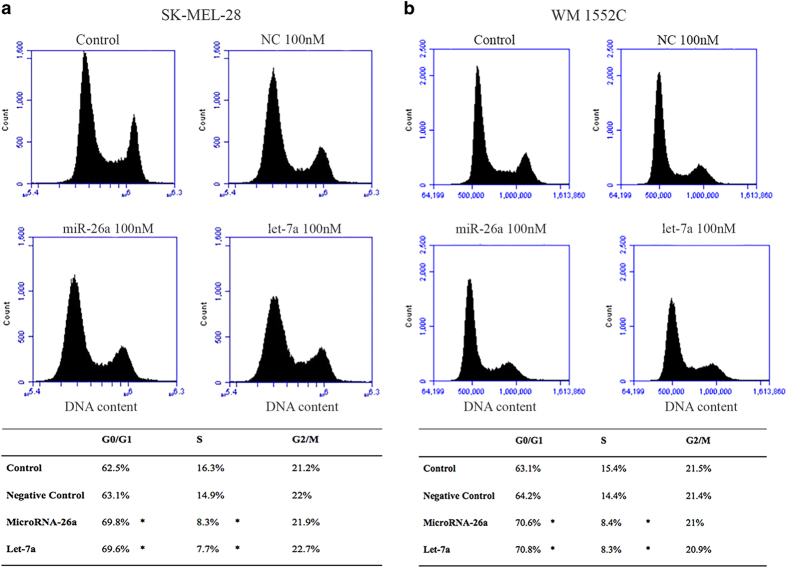
Cell cycle perturbation was measured by flow cytometry in SKMEL-28 (**a**) and WM1552C (**b**) melanoma cells. After transfection with miR-26a or let-7a (100 nM), cells were stained with PI (50 *μ*g/ml) and analyzed by flow cytometry. The percentage of cells in G_0_/G_1_, S, and G_2_/M phases was calculated using the C Flow Plus Analysis Software (Accuri Cytometers Inc., Ann Arbor, MI, USA) and summarized in the table. The histogram is representative of three independent experiments. Asterisks indicate a significant difference (*P*<0.05) compared with control groups.

We hypothesize that miR-26a and let-7a inhibit cell proliferation by inducing cell cycle arrest. In both SKMEL-28 (Figure 3a) and WM1552C cell lines (Figure 3b), transfection of miR-26a or let-7a mimics showed a significant increase in the percentage of cells in the G_1_/G_0_ phase with concomitant reduction in the percentage of cells in the S phase compared with control and negative control group. The results suggested that miR-26a and let-7a may inhibit melanoma cell proliferation by inducing cell cycle arrest at the G_1_ phase.

### MiR-26a induced apoptosis

**Figure 4:**
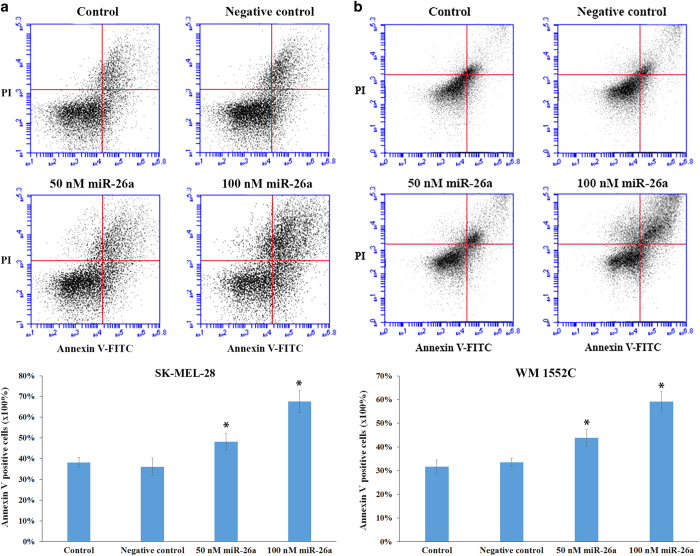
MiR-26a induced apoptosis in SKMEL-28 (**a**) and WM1552C (**b**) melanoma cells. Cells were transfected with 50 or 100 nM of miR-26a, negative control microRNA, or transfection reagent alone for 48 h. Cells were then stained with Annexin V-FITC and PI and the fluorescence intensity was measured by flow cytometry. Asterisks indicate a significant difference (*P*<0.05) from control groups.

Cell cycle arrest for an extended time can cause apoptosis, which may be an important contributor to the cytotoxicity of miR-26a. We found that transfection of miR-26a at concentrations of 50 or 100 nM led to a significant increase in the percentage of Annexin V-positive cells in both SKMEL-28 (Figure 4a) and WM1552C (Figure 4b), suggesting that miR-26a induced apoptosis in both melanoma cell lines.

### MITF is suppressed by miR-26a in melanoma cell lines

**Figure 5:**
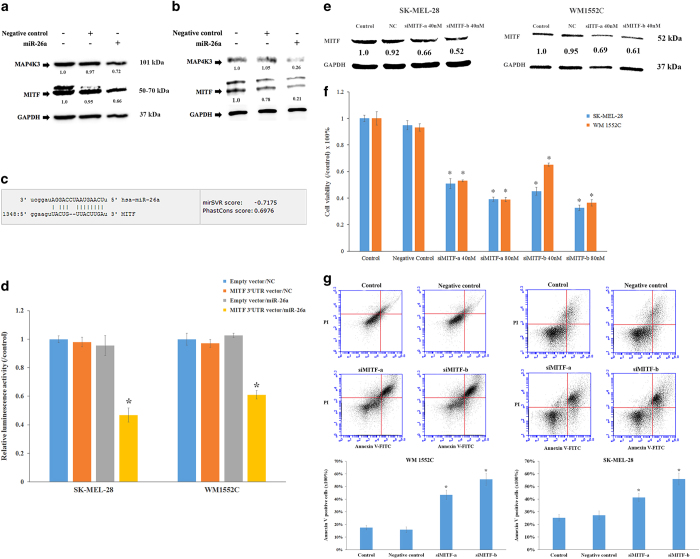
MiR-26a induced apoptosis by directly targeting the *MITF* gene. Cells were transfected with miR-26a (100 nM) or negative control for 48 h. The expressions of two hypothetical target genes, *MITF* and *MAP4K3*, were detected by western blot in SKMEL-28 (**a**) and WM1552C (**b**) melanoma cell lines. The binding sites of miR-26a on MITF 3′-UTR sequence were predicted using microRNA.org (**c**). Luciferase reporter vectors containing MITF 3′-UTR or empty vectors were used. Relative luciferase activity was significantly decreased with miR-26a co-transfection compared with the negative control for the reporter vector containing the MITF 3′-UTR but not for the empty vector (**d**). SEAP luminescence was used for normalization. Error bars represent S.D. of three replicates (*P*-value <0.01). (**e**–**g**) Knockdown of MITF reduced cell viability and induced apoptosis. (**e**) SKMEL-28 and WM1552C were transfected with siMITF-a or siMITF-b (40 nM) or negative control for 72 h, and the whole-cell extracts were subjected to western blot. (**f**) Cells were transfected with siMITF-a or siMITF-b at the concentration of 40 or 80 nM for 72 h and the cell viability was examined by MTT assay. Asterisks indicated the significant difference compared with control groups (*P*<0.05). (**g**) Cells were transfected with siMITFs (40 nM) or negative control for 72 h and then were stained with Annexin V-FITC and PI, and the fluorescence intensity was measured by flow cytometry (*P*<0.05).

Three microRNA target search algorithms (Targetscan, miRDB, and microrna.org) were used to identify hypothetical gene targets of miR-26a in human malignant melanoma. Among all predicted target genes, mitogen-activated protein kinase kinase kinase kinase 3 (MAP4K3) and MITF were chosen as the most likely targets for miR-26a. Results showed that miR-26a mimics remarkably reduced the expressions of MITF and MAP4K3 in both SKMEL-28 (Figure 5a) and WM1552C melanoma cell lines (Figure 5b).

### MiR-26a directly targets the 3′-UTR of MITF

To further investigate whether the *MITF* gene is the direct target of miR-26a in melanoma, a luciferase reporter assay was performed by using a Secrete-Pair Dual Luminescence Assay Kit (Genecopoeia Biotechnology Co., Rockville, MD, USA). The luminescence was measured after co-transfection of miRNA mimics and plasmid vectors into the two malignant melanoma cell lines. After co-transfection for 48 h, the negative control microRNA did not decrease the luciferase activity. At the same time, the transfection of miR-26a mimics did not reduce the luciferase activity produced by negative control vector without the MITF 3′-UTR sequence. However, the cells transfected with miR-26a mimics and the MITF 3′-UTR vector showed a significantly lower luciferase activity compared with the control and the negative controls (Figure 5d). The Secrete-Pair Dual Luminescence Assay (Genecopoeia Biotechnology Co.) indicated that miR-26a directly binds the 3′-UTR of the *MITF* gene, resulting in lower expression of Gaussia luciferase. Therefore, it was validated that MITF is a *bona fide* target of miR-26a.

### Knockdown of MITF reduced cell viability, and induced cell apoptosis in melanoma cell lines

To test whether miR-26a targeting MITF was responsible for the reduced cell viability observed from miR-26a mimics, we knocked down the *MITF *gene expression in both SKMEL-28 and WM1552C melanoma cell lines using siRNAs. Cell lines were treated with siRNA (40 nM) against MITF for 72 h and subjected to western blot, MTT and apoptosis assays. Both siMITF-a and siMIFT-b successfully reduced the expression of MITF (Figure 5e). In addition, both siRNAs against MIFT significantly decreased the cell viability of SKMEL-28 and WM1552C melanoma cell lines (Figure 5f). Transfection of siMITF-a or siMITF-b (40 nM) markedly increased the Annexin V-positive cell populations in both SKMEL-28 and WM1552C melanoma cells, suggesting that the knockdown of MIFT induced apoptosis in both cell lines (Figure 5g). Thus, results from both the cell viability assay and the apoptosis assay upon siMITF treatment were generally consistent with results for miR-26a treatments.

### MiR-26a retarded the growth of melanoma in mice

**Figure 6:**
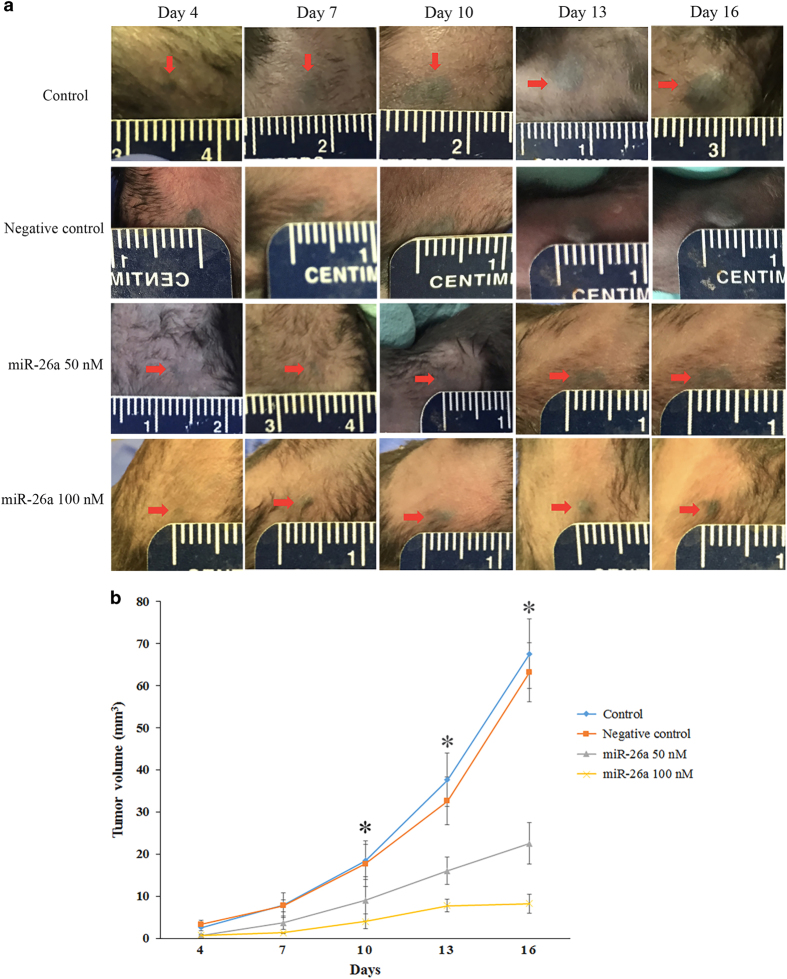
The effect of miR-26a on tumor growth in mice. B16-F10 mouse melanoma cells were transfected with miR-26a (50 or 100 nM) or negative control for 48 h, and 2×10^5^ transfected cell were subcutaneously implanted into the hint flank of male C57BL/6 mice (*n*=8) to produce tumors. Tumors were measured in 72 h intervals for 16 days. The representative tumors from different groups during the treatment course are shown in graph (**a**). Tumor volume was calculated with the following formula: (length x width^2^)/2. The tumor growth curve versus time course was plotted (**b**). Data are represented as mean±S.D. *Results comparing miR-26a transfection group and control groups at day 10, 13, and 16 were statistically significant (*P*<0.05).

While the results of the cell-based assays showed that miR-26a inhibited the growth of melanoma cells, it is important to determine whether miR-26a inhibited the tumor growth *in vivo*. B16-F10 mouse melanoma cells transfected with miR-26a (50 or 100 nM) or negative control were subcutaneously implanted into the hind flank of mice for tumor formation. Tumor size was then measured every 3 days for 16 days to observe the effect of miR-26a on tumor growth. MiR-26a at the concentrations of 50 and 100 nM significantly reduced the average size of tumors on 10th, 13th and 16th day in comparison with the control and negative control groups (Figures 6a and b), suggesting that miR-26a retarded the growth of melanoma *in vivo*.

## Discussion

Prior studies in melanoma have identified several miRNAs that are involved in melanoma progression.^28,^^29^ The inhibitory effect of miR-26a has been observed in gallbladder cancer, nasopharyngeal cancer, esophageal squamous cell cancer, and melanoma.^9,12,13,28^ Our study reported for the first time that, in malignant melanoma cells, miR-26a directly targets on MITF, which is a master regulator of melanocytes and sustains the cell viability.^29^ Therefore, miR-26a may be used to as a novel therapeutic small molecule against human malignant melanoma, possibly by regulating the expression of MITF. In addition, MITF is a potential therapeutic target for melanoma treatment. The inhibitory effect of let-7a has been observed in human papillomavirus-induced cervical carcinogenesis and Burkitt lymphoma cells.^30,31^ Previous research also found that let-7a induced apoptosis in various cancers.^19,20,21,25^ However, the role of let-7a on melanoma has been largely unknown. We report for the first time that a let-7a mimics significantly reduced the cell viability in several melanoma cell lines.

We showed that miR-26a and let-7a decreased the cell viability by causing a cell cycle arrest at the G1/S phase. This mechanism is in agreement with ability for miR-26a to arrest the cell cycle in ACTH-secreting pituitary adenomas, lung carcinoma^32^ and hepatocellular carcinoma.^33^ It is also consistent with the findings that let-7a arrests the cell cycle of human hepatocellular carcinoma.^19,34^ In addition, the miR-26a and let-7a mimics decreased the ability of malignant melanoma cells to migrate across the Transwell membrane, suggesting that miR-26a and let-7a may inhibit invasiveness and metastasis of melanoma. Similar results were found in nasopharyngeal cancer, gastric cancer, esophageal squamous cell cancer, and pancreatic cancer cells where miR-26a inhibited metastasis.^28,35,36^ Similarly, let-7a has been shown to inhibit metastatic properties of nasopharyngeal carcinoma, gastric cancer, breast cancer, and lung cancer.^14,17,37,38^

Several genes have been identified and validated to be the targets of miR-26a in multiple cancers including melanoma.^12,13,39^ Reuland *et al*.^13^ reported that the expression of miR-26a is significantly downregulated in melanomas compared with melanocytes and miR-26a mediates apoptosis in melanoma by targeting the SODD in a few sensitive cell lines of melanoma.^13^ This study reports that MITF is a *bona fide* target of miR-26a. MITF has an important role in melanoma initiation and development due to its oncogenic function specifically for the melanocytes.^40^ MITF drives the progression of melanomas harboring BRAF^V600E^ mutation by promoting melanocytes to synthesize the melanin to retain melanoma and activating the overexpression of antiapoptotic genes for survival.^41^ Therefore, MITF is a potential novel therapeutic target for malignant melanoma, and miR-26a may exert its antimelanoma effects by targeting MITF. It is also worth mentioning that miR-26a targets other genes including SODD and SMAD1. Therefore, like any other tumor suppressor microRNA, the inhibitory effect of miR-26a may be the combinatorial effect of targeting several oncogenic genes. In addition, this study showed that miR-26a reduced the expression of MAP4K3 in both melanoma cell lines. MAP4K3 is a Ser/Thr kinase that is believed to regulate the activation of mTOR pathway,^42^ which is crucial in regulating cell proliferation, cell growth, and inducing autophagy.^43^ A variety of cancers can be caused by hyperactivation of the mTOR pathway.^44–46^ Owing to the importance of mTOR pathway and the major role of MAP4K3 in activating mTOR pathway, miR-26a may inhibit cell viability and invasiveness by downregulating MAP4K3.

In conclusion, miR-26a and let-7a showed several strong anticancer properties. Both microRNAs significantly decreased the proliferation and invasion of melanoma cells. Along with targeting other oncogenic target genes such as SODD, SMAD1, and MAP4K3, miR-26a inhibited the growth of melanoma cells by directly downregulating MITF. We have found that miR-26a and let-7a act as tumor suppressors in melanoma cells, and replacement therapy with miR-26a and let-7a represents a promising novel therapeutic strategy against human malignant melanoma.

## Materials and methods

### Cell culture, miRNA, and siRNA transfection

The human malignant melanoma cell line WM1552C (The Wistar Institute, Philadelphia, PA, USA) was cultured in MCDB 153 medium (Sigma, St Louis, MO, USA) supplemented with 2% fetal bovine serum (FBS; Hyclone, Logan, UT, USA) and 0.1% penicillin/streptomycin (Fisher Bioreagents, Pittsburgh, PA, USA). The melanoma cell line SKMEL-28 (ATCC, Manassas, VA, USA) was cultured in Eagle’s minimum essential medium (ATCC, Manassas, VA, USA) supplemented with 10 FBS and 0.1% penicillin/streptomycin. Both cell lines were cultured at 37 °C in a 5% CO_2_ atmosphere incubator (Thermo scientific, Pittsburgh, PA, USA) with humidity. The mouse melanoma cell line B16-F10 was maintained in Dulbecco’s modified Eagle’s medium (Sigma, St Louis, MO, USA) supplemented with 10% FBS. The hsa-let-7a-5p mimic has a mature miRNA sequence of 5′- UGAGGUAGUAGGUUGUAUAGUU-3′, and the miR-26a-5p mimic has a mature miRNA sequence of 5′- UUCAAGUAAUCCAGGAUAGGCU-3′. The hsa-let-7a-5p (MSY0000062), miR-26a-5p (MSY0000082) and AllStars negative control siRNA were purchased from Qiagen (Germantown, MD, USA) and transfected into the cells using DharmFECT transfection reagent 2 (Thermo Scientific, Pittsburgh, PA, USA) at a final concentration of 50 or 100 nM. FlexiTube siRNAs (Qiagen) specific to MITF were used for knockdown of the *MITF* gene as per the manufacturer’s instructions. Two siRNAs specific to *MITF* gene were used to guard against the possibility of off-target effects. These were Hs_MITF_1 (cat. no. SI00005362) and Hs_MITF_5 (cat. no. SI02781212). These siRNAs are referred to hereafter as siMITF-a and siMITF-b, respectively. Cells were transfected with siRNAs (40 nM) using HiPerFect (QIAGEN Inc., Valencia, CA, USA). AllStars negative control siRNA was also used as a control. Both MTT and Annexin V apoptosis assays were conducted 72 h after transfection.

### MicroRNA isolation and quantitative RT-PCR

Total microRNA was isolated from WM1552C and SKMEL-28 human melanoma cell lines after transfection for 48 h using mirVana miRNA Isolation Kit (Life Technologies, Carlsbad, CA, USA) as per the manufacturer’s instructions. A NanoDrop 2000C Spectrophotometer (Thermo Scientific, Wilmington, DE, USA) was used to assess the quality and measure the concentration of the isolated microRNA. Two step reactions were performed by using Universal cDNA Synthesis Kit (Exiqon, Woburn, MA, USA) and SYBR Green Master Mix PCR Kit (Exiqon) for the cDNA synthesis and PCR, respectively. U6 snRNA served as an endogenous control.

### Cell viability assay

Cells were transfected with miR-26a mimics, let-7a mimics, and negative control mimics at a final concentration of 50 and 100 nM immediately after being seeded into 96-well plate (Corning Costar, Cambridge, MA, USA). After 48 h incubation period, 10 *μ*l MTT (3, (4, 5-dimethylthiazol-2-yl)-2, 5-diphenyltetrazolium bromide) reagent (Trevigen, Gaithersburg, MD, USA) was added into each well and detergent solution was applied 2 h later to dissolve the insoluble purple formazan. The absorbance of solubilized dye was measured by a microplate reader (BioTek Instrument, Winooski, VT, USA) at 570 nm. Six independent experiments were performed and the results were reported as the mean±S.D.

### Wound healing assay

Cells were trypsinized after 48 h transfection and seeded into a 24-well plate (Corning Costar, Cambridge, MA, USA) with 90% confluency. An artificial wound was created with a 200 *μ*l pipette tip (Fisherbrand, Pittsburgh, PA, USA) in the center of the confluent cell monolayer. PBS solution was used to wash off cell debris in each well and the cells were incubated for additional 24 h. The closure of the wound in each group was evaluated under an inverted phase contrast microscope (×40).

### Cell invasion assay

Cells (1×10^5^) in 400 *μ*l serum-free medium were plated into the top chamber of 24-well Transwell inserts (Corning Incorporated, Corning, NY, USA), which has a Matrigel-coated membrane with 8-*μ*m pores. In the bottom chamber, 10% FBS in normal medium was added to serve as a chemoattractant. MTT reagent was applied to each well after 24 h incubation for cell staining. Cells on the top side of the chamber were removed by a cotton swab and the invasive cells on the bottom side of the membrane were evaluated with an inverted microscope. For colorimetric quantification, wells were filled with 200 *μ*l DMSO for 20 min in the dark to dissolve formazan. Then, dissolved dye in DMSO was moved to 96-well plate and the absorbance was measured using a microplate reader at 570 nm.

### Cell cycle analysis

Cells were harvested 48 h after transfection, washed with cold PBS, and fixed in 90% ice-cold ethanol for 1 h at room temperature. Cells were then washed with cold PBS and centrifuged at 100×*g* for 5 min and resuspended in 500 *μ*l propidium iodide (PI, 50 *μ*g/ml) in the dark. After 1 h, cells were incubated with 100 *μ*l RNase (100 *μ*g/ml) for additional 30 min in 37 °C before measuring DNA content by using Accuri C6 Flow Cytometer System (Accuri Cytometer Inc., Ann Arbor, MI, USA). Populations in G_0_/G_1_, S and G_2_/M phases were shown as percentage of total gated cells. The measurements were performed by three independent experiments.

### Apoptosis analysis by flow cytometry

Apoptosis was examined using an Annexin V-FITC-PI Dual Staining Kit (Biolegend, San Diego, CA, USA) followed by flow cytometry analysis as per the manufacturer’s instructions. Briefly, SKMEL-28 and WM1552C cells were transfected with miR-26a mimics (50 or 100 *μ*M) or negative control microRNA mimics for 48 h and harvested by trypsinization, washed with ice-cold PBS, and resuspended in binding buffer at a density of 1×10^6^ cells per ml. Cell suspension was stained with Annexin V and PI and analyzed by the Accuri C6 Flow Cytometer System.

### Western blot analysis

After 48 h transfection, cells were trypsinized and washed three times with PBS and then lysed in lysis buffer for 30 min on ice. The protein was then electrophoresed on an AnyKD SDS-PAGE gel (Bio-Rad, Hercules, CA, USA) and then transferred to a nitrocellulose membrane (Bio-Rad) and blocked with 5% non-fat milk in TBST for 2 h at room temperature with shaking. The membrane was immunoblotted with primary antibody for rabbit anti-GAPDH, anti-MITF and anti-MAP4K3 (Cell Signaling Technology, Danvers, MA, USA) with dilutions of 1 : 1000, 1 : 1000 and 1 : 1000, respectively, at 4 °C overnight. Signals were developed using HRP-linked secondary antibody (1 : 10 000) with Clarity Western ECL Substrate (Bio-Rad). The intensity of the signals was determined by FluorChem E system (Protein Simple, Santa Clara, CA, USA).

### Plasmid construction and dual luminescence assay

Secrete-Pair Dual Luminescence Assay (Genecopoeia Biotechnology Co.) was applied to investigate whether miR-26a directly regulates the expression of MITF in two malignant melanoma cell lines. The recombinant MITF vector, containing MITF 3′-UTR that contains miR-26a binding site was purchased from GeneCopoeia (Rockville, MD, USA). The recombinant control vector without the MITF 3′-UTR was used as a negative control vector. MiR-26a mimics or negative control mimics at a final concentration of 100 nM were co-transfected with the MITF vector or a negative control vector (1 *μ*g/*μ*l) using the TransIT-X2 Dynamic Delivery System (Madison, WI, USA) into SKMEL-28 and WM1552C melanoma cells. After 48 h transfection, cells were trypsinized and the luminescence assay was performed by using Secrete-Pair Dual Luminescence Assay Kit (Genecopoeia Biotechnology Co.) according to the manufacturer’s instructions. Luminescence activity was measured by a microplate reader.

### *In vivo* tumor growth model

All animal protocols were approved by Animal Care and Use Committee of Emporia State University. Six-week-old male C57BL/6 mice were obtained from Harlan Laboratories Inc. (Indianapolis, IN, USA) and housed in controlled animal facility with 12 h light–dark cycle and allowed to have *ad libitum* access to rodent pellet and fresh tap water. A total of 32 mice were randomly divided into four groups (eight mice per group). Cells were transfected with miR-26a at concentrations of 50 or 100 nM, or with AllStars negative control siRNA for 48 h. A total of 2×10^5^ transfected cells were subcutaneously implanted into the hind flank of mice to produce tumor. The size of the tumor was measured every 3 days for 16 days. Tumor volumes were calculated as (width)^2^×length/2.

### Statistical analysis

All values were represented as the mean±S.D. from at least three independent experiments. Statistical difference between groups was measured using the Student’s *T*-test. *P*-value in all experiments were considered significant at ⩽0.05.
