# Supplementary figures and images for: MicroRNA-26a inhibits the growth and invasiveness of malignant melanoma and directly targets on MITF gene
Source: Cell Death Discov. 2017 Jul 10;3:17028–. doi: 10.1038/cddiscovery.2017.28 (PMC5502303; doi:10.1038/cddiscovery.2017.28)

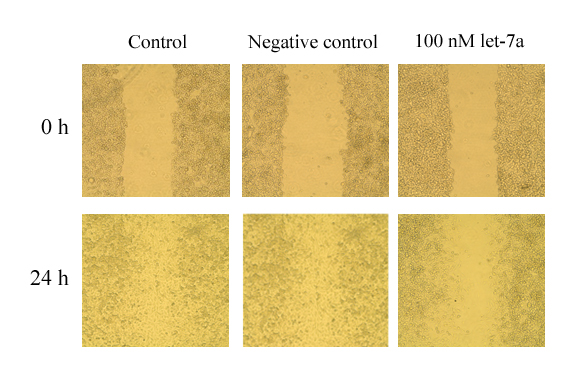

Supplement: Supplementary Figure 1 [file cddiscovery201728-s1.jpg]
