# Supplementary material for: MicroRNA-26a inhibits the growth and invasiveness of malignant melanoma and directly targets on MITF gene
Source: Cell Death Discov. 2017 Jul 10;3:17028–. doi: 10.1038/cddiscovery.2017.28 (PMC5502303; doi:10.1038/cddiscovery.2017.28)
Supplement: Supplementary Figure Legend [file cddiscovery201728-s2.doc]

Supplementary figure 1. After 48 h transfection of the negative control, microRNA mimics or let-7a mimics at the final concentration of 100 nM, the cell migration assay was performed as described in the Materials and Methods section. The closure of the wound in each group was evaluated under an inverted phase contrast microscope (40X).
